# Supplementary material for: Skin-Grafting and Dendritic Cell “Boosted” Humanized Mouse Models Allow the Pre-Clinical Evaluation of Therapeutic Cancer Vaccines
Source: Cells. 2023 Aug 18;12(16):2094. doi: 10.3390/cells12162094 (PMC10453599; doi:10.3390/cells12162094)
Supplement: Supplementary file 1 [file cells-12-02094-s001.zip › cells-2489743-supplementary.pdf]

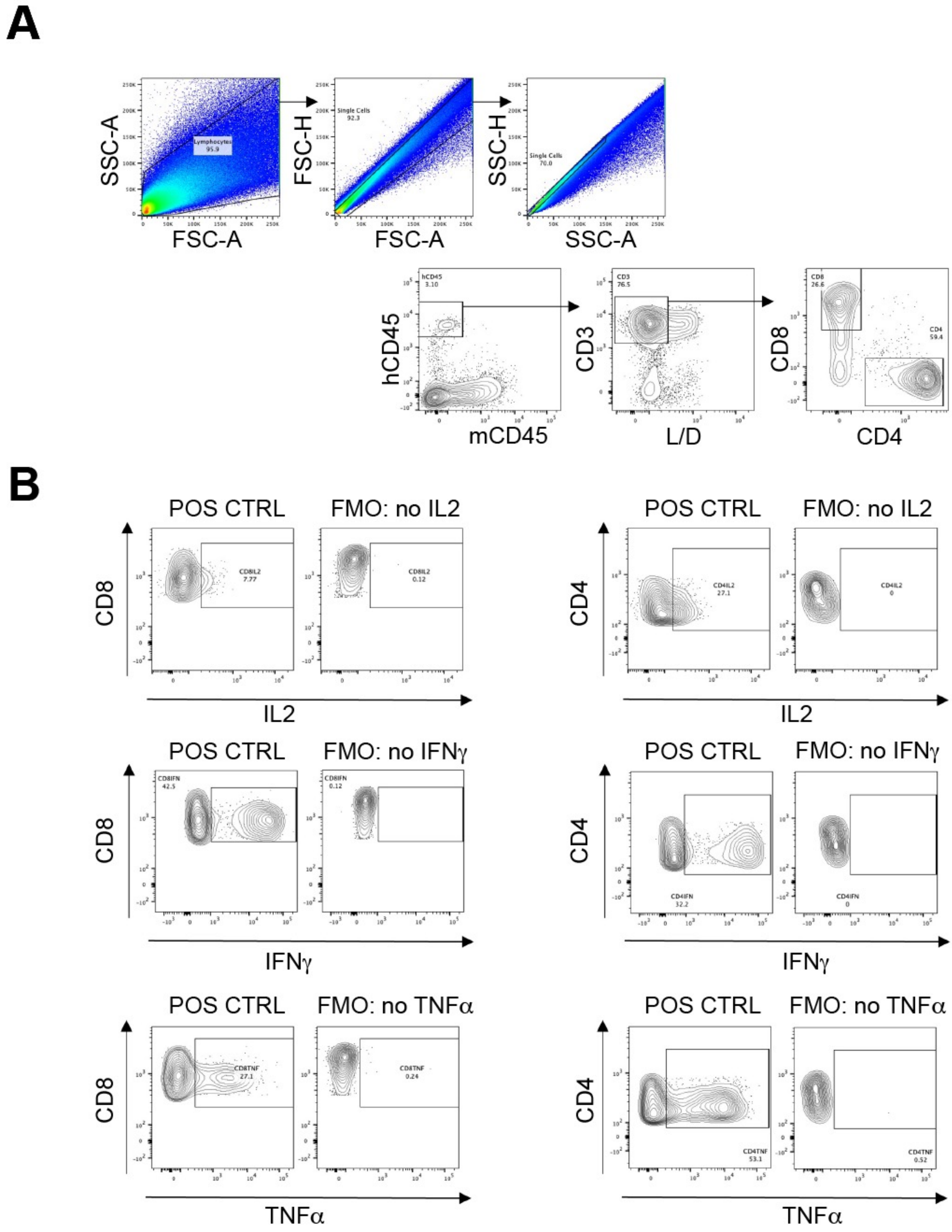

Figure S1. Gating strategy for the polyfunctional assay. (A) Physical parameters are used to exclude doublets. $hCD45^+$ lymphocytes are then separated from $mCD45^+$ lymphocytes and $CD8^+$ and $CD4^+$ T cells are gated within $hCD45^+CD3^+$ cells. **(B)** Human $CD8^+$ and $CD4^+$ T cells further analyzed for the production of IL2, TNFα and IFNγ. Fluorescence Minus One (FMO) are used as negative control to set up gating.

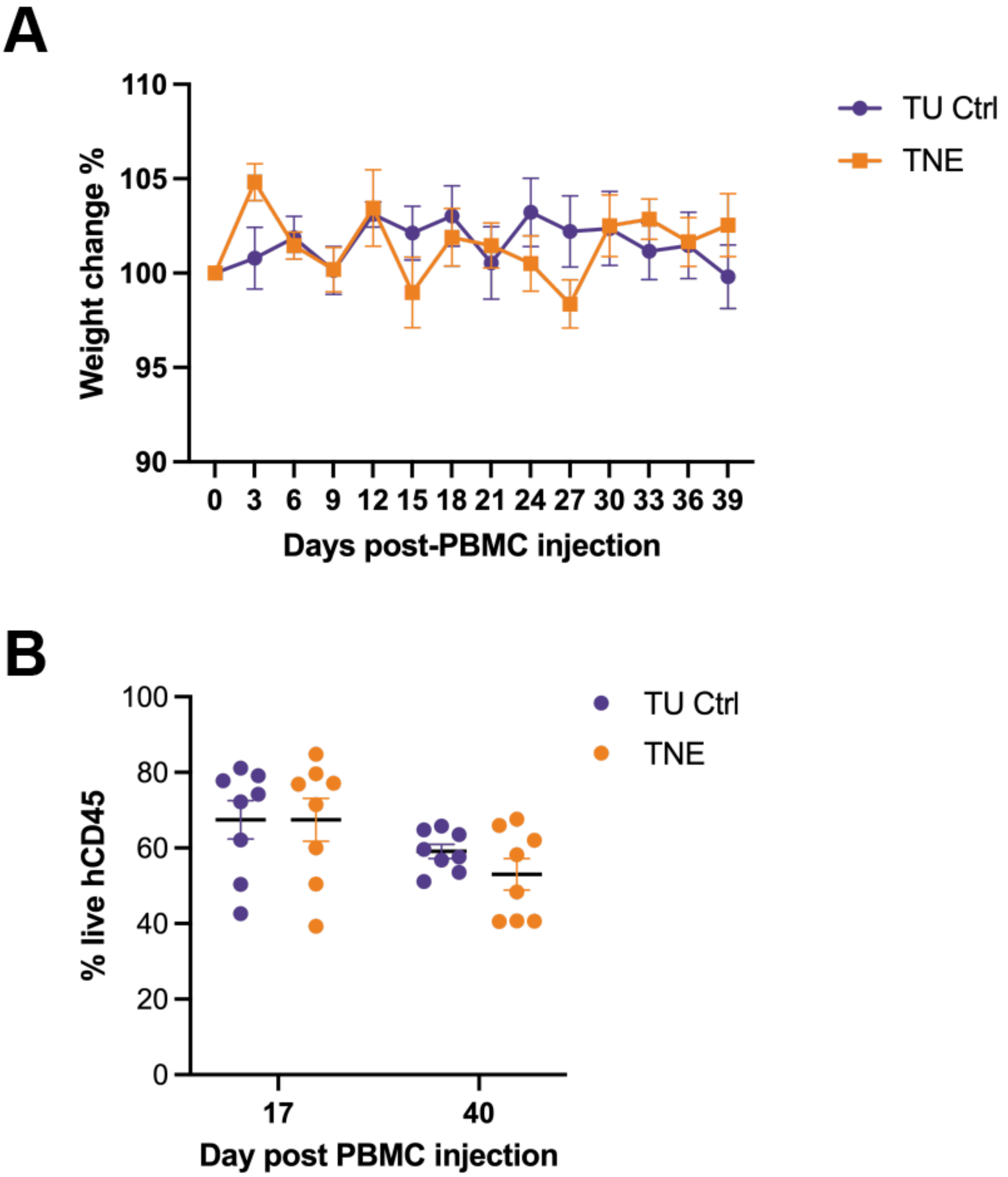

Figure S2. Boosted PBMC-hu-NSG-A2 model. (A) Body weight of mice in figure 4C & 4D expressed as the percentage of the initial weight. Signs of GvHD are typically manifested when 25% body weight loss is reached. **(B)** Engraftment of human CD45^{+} cells in the blood of mice in figure 4C & 4D was analyzed by flowcytometry at day 17 and day 40 after PBMCs injection.
